# Future Paths in Cryogenic Single-Molecule Fluorescence Spectroscopy

**DOI:** 10.1021/acs.jpcc.3c06564

**Published:** 2023-12-22

**Authors:** Subhasis Adhikari, Robert Smit, Michel Orrit

**Affiliations:** Huygens−Kamerlingh Onnes Laboratory, Leiden University, 2300 RA Leiden, The Netherlands

## Abstract

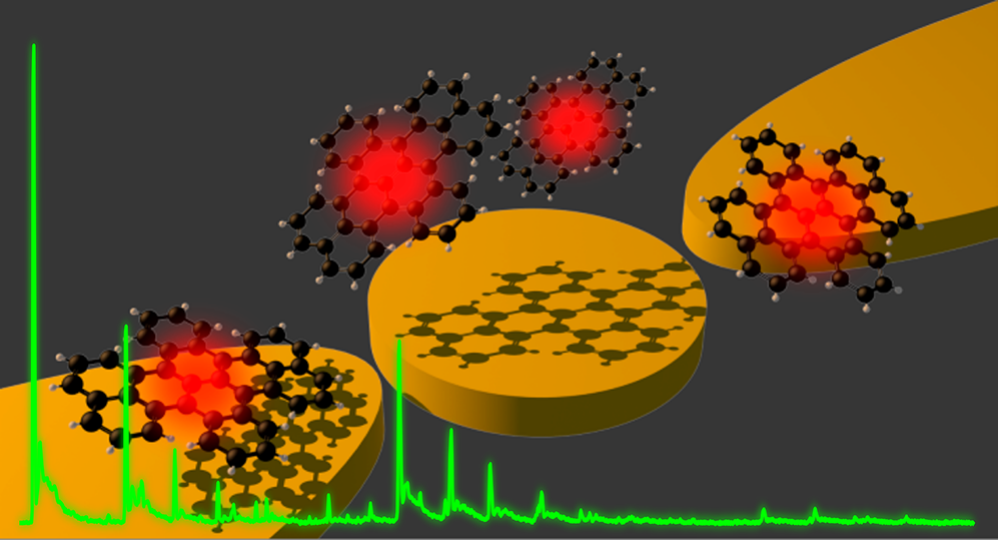

In the last three decades, cryogenic single-molecule fluorescence spectroscopy has provided average-free understanding of the photophysics and of fundamental interactions at molecular scales. Furthermore, they propose original pathways and applications in the treatment and storage of quantum information. The ultranarrow lifetime-limited zero-phonon line acts as an excellent sensor to local perturbations caused either by intrinsic dynamical degrees of freedom, or by external perturbations, such as those caused by electric fields, elastic and acoustic deformations, or light-induced dynamics. Single aromatic hydrocarbon molecules, being sensitive to nanoscale probing at nanometer scales, are potential miniaturized platforms for integrated quantum photonics. In this Perspective, we look back at some of the past advances in cryogenic optical microscopy and propose some perspectives for future development.

## Introduction

Since the first detection of a single molecule through its fluorescence in 1990,^1^ more than 30 years ago, single-molecule fluorescence spectroscopy has spread to numerous fields of research in materials science, soft-matter studies, nanophotonics, and molecular cell biology. Although early single-molecule studies were mainly devoted to fundamental studies of single fluorescent molecules in frozen matrixes, later work has broadened the scope of the method by extending it to ambient conditions, in particular to complex systems of biological molecules.^2^ The revolution of super-resolution fluorescence microscopy has enabled researchers to investigate biomolecules at a much smaller scale, close to those accessible through electron microscopy. Single-molecule fluorescence resonance energy transfer (smFRET) has become a standard tool in investigating dynamic interactions and conformational heterogeneity in biomolecules. Single-molecule fluorescence spectroscopy has uncovered and enabled probing of spatiotemporal heterogeneity at nanometer scales, beyond ensemble averaging.^3^ Meanwhile, along with room temperature single-molecule studies, considerable progress has been made in cryogenic fluorescence microscopy, the focus of this Perspective.

The first single-molecule detection^4^ was achieved at a cryogenic (liquid-helium) temperature by detection of the absorption signal of a single pentacene molecule in a solid crystal of *para*-terphenyl by dual-frequency modulation spectroscopy. Subsequent studies of single molecules through fluorescence detection were also done with the same host–guest system and at liquid-helium temperature but by fluorescence excitation, which provided a much better signal-to-noise ratio by efficiently removing background. Selection of single molecules in the difficult conditions of a liquid-helium cryostat is made possible thanks to two crucial advantages of cryogenic temperatures. The first one is that single molecules at cryogenic temperatures are no longer perturbed by thermal fluctuations; therefore, their red-most absorption line (called the zero-phonon line, ZPL) becomes extremely narrow. The interaction of the molecule with a properly tuned single-frequency laser is therefore enhanced by several orders of magnitude. The second advantage is that photoinduced chemical reactions are practically blocked at low temperature, so that a single fluorescent molecule can provide nearly unlimited numbers of fluorescence photons, free from photoinduced damage or photobleaching. In many ways, single bright and photostable fluorescent molecules at a cryogenic temperature behave as ideal individual two-level systems and are excellent single-photon sources for integrated quantum photonics.^5^ Cryogenic single-molecule fluorescence spectroscopy has strongly focused on quantum-optical applications and single-photon interactions, notably those requiring coherent interaction and indistinguishable photons.^6−8^ The photons emitted on the lifetime-limited ZPL of a single fluorescent molecule are indistinguishable and can be spectrally tuned by external perturbations such as electric field,^9^ elastic deformations,^10,11^ and optically induced perturbations.^12^ Photons hardly interact with one another, which makes them attractive carriers to transmit quantum information. Many quantum-optical protocols, however, require such photon–photon interactions, which are much harder to achieve than with electrical charges.^13,14^ A single quantum system such as a single molecule can act as an efficient coupler of photons. For example, a molecule excited by a first photon will become transparent to a second photon. Plasmonic nanostructures,^15^ optical microcavities,^16,17^ and plasmonic/dielectric waveguides^9,18^ can further amplify photon–photon interactions in single molecules.

Single fluorescent molecules are excellent nanoscale probes for investigating spatial and temporal heterogeneities, which can occur even in a solid matrix at cryogenic temperatures. Crystal defects, lattice distortions, domain walls, and polycrystalline subdomains all can affect the local environment of single molecules. Such defects can be intrinsic to the host matrix or induced by the insertion of impurities or guest molecules in the host. Several earlier single-molecule studies^19−24^ reported a broad distribution of ZPL spectral line widths and temporal changes in ZPL spectral positions, giving rise to spectral diffusion. Spectral diffusion or spectral jumps of the ZPL are often caused by switching between states of one or more two-level systems (TLS). Even though most dynamic processes are frozen at cryogenic temperatures by the lack of activation above most energy barriers, jumps between TLSs may still occur, owing to their low activation energy barriers. Spectral diffusion leads to the intermittency or photoblinking of a fluorescence signal. Photoblinking can also occur due to non-radiative intersystem crossing (ISC) to the triplet state or to other dark states.^25^ Therefore, a guest–host system needs to fulfill a number of conditions to be practically useful in cryogenic single-molecule fluorescence experiments.

In this Perspective, we will briefly discuss past and recent developments in cryogenic single-molecule fluorescence spectroscopy with a major focus on studies from the past few years. In addition, we will mention some possible future developments that we discuss in more detail.

## Past and Recent Developments in Cryogenic Single-Molecule Fluorescence Spectroscopy

Earlier single-molecule studies were devoted to investigating new guest–host systems and understanding their spectral properties. For cryogenic experiments, an ideal guest–host system fulfills the following requirements for the guest molecules: (i) (near) lifetime-limited ZPL, (ii) strong absorption cross-section, (iii) large Debye–Waller factor, (iv) low ISC rate, (v) high fluorescence quantum yield, (vi) (near) absence of fluorescence intermittency, (vii) (near) absence of spectral diffusion, and (viii) highly photostable. Mostly, aromatic fluorescent molecules such as pentacene, perylene (Pr), terrylene (Tr), dibenzoterrylene (DBT), and dibenzanthanthrene (DBATT) have been used as guest molecules inside host matrixes such as Shpol’skii matrixes of linear alkanes, polymers, crystals such as naphthalene, anthracene, and *para*-terphenyl. Some guest–host systems such as DBT in anthracene^26^ or DBATT in *n*-tetradecane^27^ are close to ideal and have vastly been used for applications in integrated quantum photonics. We first briefly discuss the past developments and then focus on more recent results.

**Figure 1:**
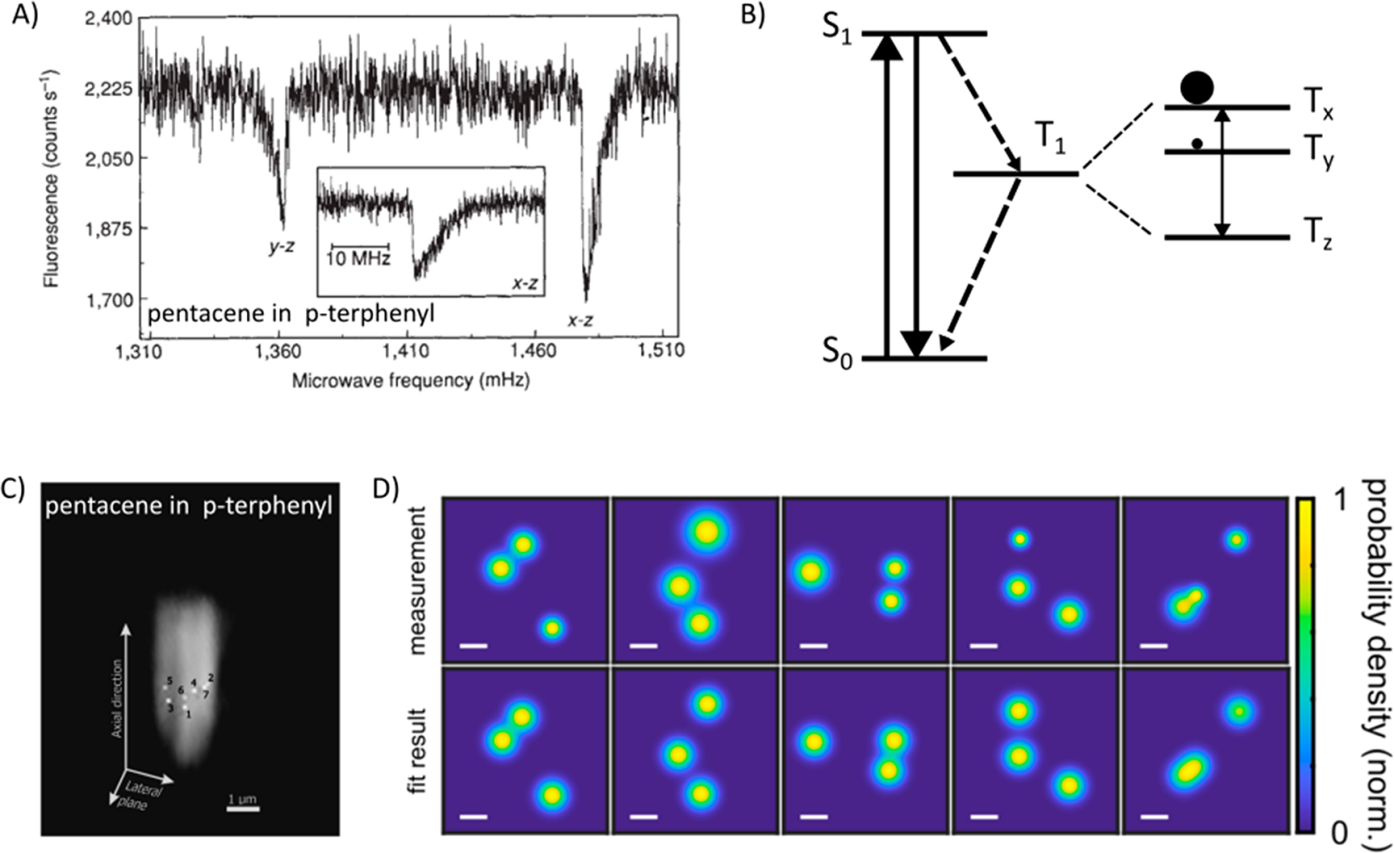
(A) Decrease in the signal of a single fluorescent molecule when applying microwaves resonant with T_*y*_ → T_*z*_ and T_*x*_ → T_*z*_ transitions (inset with lower microwave power), indicating the magnetic resonance of a single molecular electron spin. Reprinted with permission from ref (36). Copyright 1993 Springer Nature Limited. (B) Schematic representation of a three-level energy diagram of a pentacene molecule (S_0_, S_1_, and T_1_ states). Solid and dashed arrows indicate radiative or radiation-less transitions. The three triplet states (T_*x*_, T_*y*_, and T_*z*_) split by the magnetic dipole–dipole interaction are shown on the right-hand side, and their respective population probabilities are indicated by the solid circles. (C) Super-resolution microscopy of single fluorescent molecules (7 bright dots) with their three-dimensional intensity distributions at low temperature, taking advantage of combined spatial localization and spectral selection. Reprinted with permission from ref (37). Copyright 1999 The Optical Society. (D) Super-resolution images of single proteins labeled with ATTO647N fluorophores (top and bottom panels showing experimental and simulated images, respectively). The color codes represent the localization probability densities normalized to their maximum. Reprinted with permission from ref (39). Copyright 2022 eLife.

Earlier cryogenic single-molecule fluorescence experiments^24,28^ on pentacene molecules in a *p*-terphenyl crystal had found lifetime-limited ZPLs, while antibunching of fluorescence photons indicated single fluorescent molecules to be efficient single-photon sources. Furthermore, it was demonstrated that the narrow ZPL could be spectrally tuned by applying hydrostatic pressure^29^ or an external electric field via linear or quadratic Stark effect.^30^ Orrit’s group reported the Stark effect of single molecules in several guest–host systems.^31−34^ One of the milestones in earlier single-molecule studies was the optical detection of the electron spin of a single pentacene molecule in a *p*-terphenyl crystal using combined optically detected magnetic resonance (ODMR) and single-molecule fluorescence spectroscopy^35,36^ (Figure 1A and B). By applying a resonant microwave frequency and observing the change in the fluorescence signal of a single molecule, the transitions between different sublevels of a triplet state can be detected. The population and relaxation channels of states T_*x*_, T_*y*_, and T_*z*_ are schematically shown in Figure 1B. Single-molecule ODMR measurements opened a new way to study magnetic resonance down to the single-molecule level, which was later extended to single color centers in diamond. An earlier demonstration of far-field super-resolution beyond the diffraction limit was first reported at cryogenic temperatures for a single pentacene molecule in a *para*-terphenyl matrix with an accuracy of 40 nm^37^ (Figure 1C). Combining position-sensitive imaging with spectral selection allowed researchers to localize several molecules within a diffraction-limited volume. Later, cryogenic single-molecule studies achieved an accuracy down to Angström resolution^38^ by taking advantage of high molecular photostability at a cryogenic temperature (Figure 1D).

**Figure 2:**
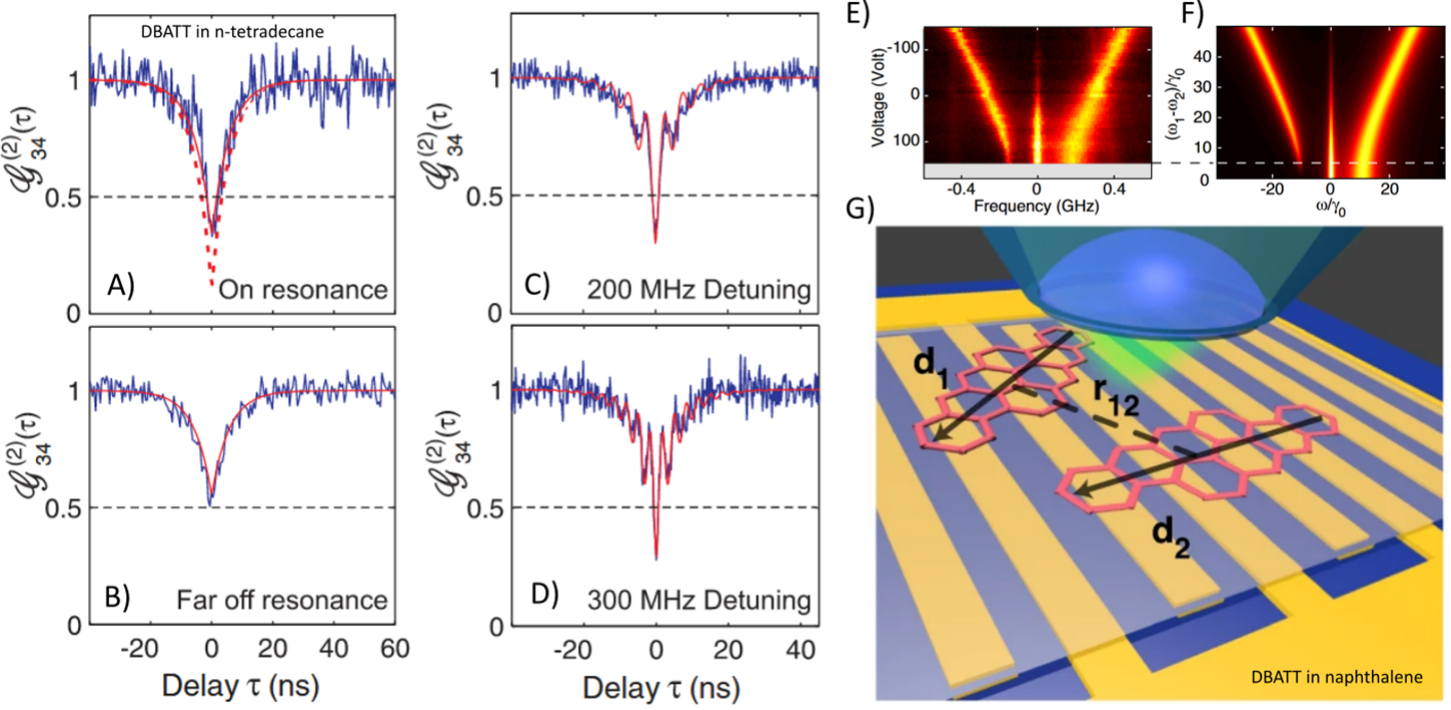
(A) Intensity cross-correlations of two photons emitted by two resonant single molecules, i.e., two molecules with ZPLs at the same frequency. (B) Off-resonant molecules, i.e., the ZPL of one molecule is detuned by 5 GHz with respect to the other molecule. (C) ZPLs detuned by 200 MHz and (D) ZPLs detuned by 300 MHz. Reprinted with permission from ref (27). Copyright 2010 American Physical Society. (E) Experimental and (F) simulated spectral trails of sub- and super-radiant energy states arising from coherent coupling of two single molecules which are spectrally tuned by the Stark effect. (G) Schematic representation of two DBATT molecules (size exaggerated) doped in a naphthalene crystal over a micropatterned gold electrode to apply the electric field. The molecules are illuminated with a high-NA objective, which also collects the fluorescence signal. Reprinted with permission from ref (49). Copyright 2022 Springer Nature.

Sandoghdar’s group^40^ first demonstrated a coherent dipole–dipole coupling between two single molecules that were a few nanometers apart and entanglement of photons from two molecules, creating sub- and super-radiant energy states. This was an early step toward the integration of single molecules as nanometer-sized elements into quantum photonic circuits.^41^ For applications of single molecules in the optical treatment of quantum information, the interaction between indistinguishable photons is a prerequisite. Later experiments^42^ demonstrated two-photon interference from a single molecule, which proved that single molecules are capable of emitting indistinguishable photons. Several articles reported coherent manipulations of single-photon interactions^43,44,27,45−48^ (see one example in Figure 2A–D), and a recent review article summarizes this work.^6^

**Figure 3:**
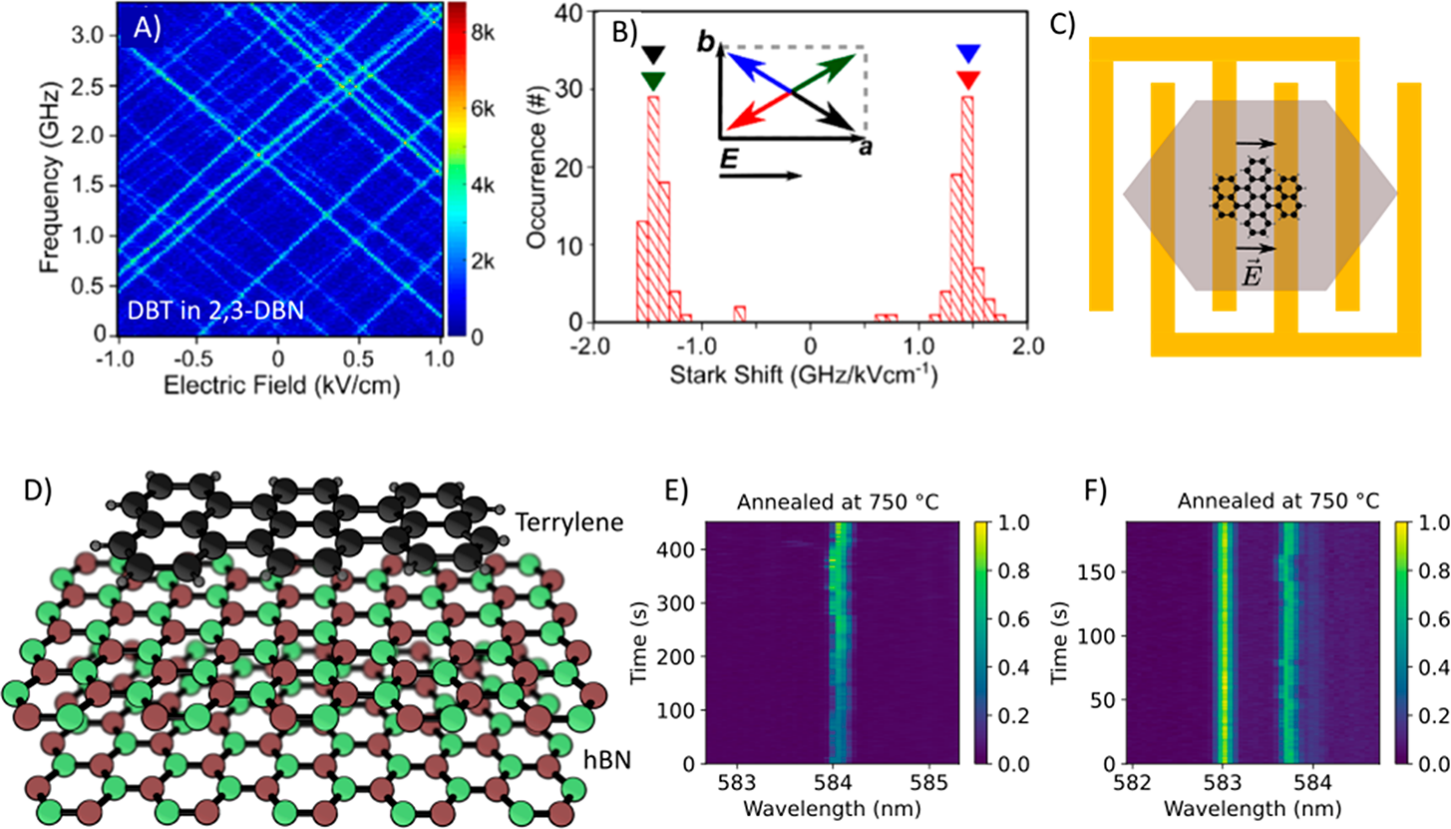
(A) Linear Stark shift of single DBT molecules in a DBN crystal and (B) distribution of Stark coefficients. The inset in (B) gives the orientations of the four possible molecular dipoles with respect to the crystal axes and to the applied electric field. Reprinted with permission from ref (33). Copyright 2019 John Wiley and Sons. (C) Schematic of a DBT molecule embedded in a DBN crystal on top of a gold electrode. (D) Chemical structures of terrylene and hexagonal boron nitride (hBN). (E and F) ZPLs of single terrylene molecules on the surface of hBN showing high spectral stability. In (E), the laser power was increased every 100 s. Samples were annealed at 750 °C. Reprinted with permission from ref (58).

For a controlled coherent interaction of single photons with single fluorescent molecules, spectral tuning is necessary. The ultranarrow ZPL of a single molecule can be reversibly tuned by varying the external electric field or elastic strain or irreversibly varied by optically pumping with a strong laser.^12^ The phenomenon of a spectral shift tuned by an external electric field is known as the Stark effect. There are two types of Stark effect, the so-called linear and quadratic Stark effects, which respectively depend linearly and quadratically on the applied electric field. The linear Stark effect is often larger than the quadratic effect and requires much weaker electric fields for spectral tuning. For a centrosymmetric molecule, the Stark effect is, in general, quadratic due to the absence of a net permanent dipole moment. However, inside a host matrix, due to crystal-induced symmetry breaking, some molecules can gain a net permanent dipole moment and show a linear Stark effect. Recently, Moradi et al.^33^ demonstrated a strong linear Stark effect for a centrosymmetric DBT molecule inside a 2,3-dibromonapthalene (DBN) host matrix, with a Stark coefficient of about 1.5 GHz/kV cm^–1^ (Figure 3A–C). One of the remarkable results of this study was that almost all molecules showed similar Stark coefficients, in contrast to the broad distributions of Stark coefficients found in other guest–host systems. This new host–guest system is thus very promising for probing single-charge dynamics inside (or outside) a solid matrix. The linear Stark effect has been observed in the same system by laser-induced tuning,^12^ instead of an external electric field. A highly focused strong pump laser induced photoionization in the host matrix, which separated charge carriers that were locally trapped by defects. These charges created an internal electric field, which locally shifted the guest molecules’ spectral line via the Stark effect. This all-optical approach for frequency tuning, which can tune molecules in resonance with each other, is potentially useful for applications in fast quantum nanophotonics. The Stark effect was also used for a coherent manipulation^49^ of two coupled dye molecules, several tens of nanometers apart. The molecules were localized using excited-state saturation (ESSat) nanoscopy, a far-field super-resolution technique recently developed by Lounis and co-workers.^50^ Using a hyperspectral imaging method, they have provided direct evidence of coherent dipole–dipole interaction creating sub- and super-radiant energy states (Figure 2E–G). Fluorescence lifetime measurements showed that subradiant energy states were long-lived (11.1 ns) compared to uncoupled molecules (7.8 ns), whereas super-radiant states decayed faster (6.3 ns). The ZPL line width of the subradiant energy state (∼13 MHz) was found to be narrower than the natural line width (∼23 MHz). This study broadens the coherent manipulation of entanglement between two distant molecules. Coherent coupling is, in general, hampered by incoherent vibrational coupling. To enhance the coherent coupling, a fluorescent molecule could be coupled to an optical microcavity.^16^ A remarkable achievement of coherent coupling was obtained between a molecule in a microcavity and single photons that were generated from a distant molecule in a different laboratory. Coherent coupling between a plasmonic nanoparticle and a molecule was also observed at cryogenic temperatures^15^ and led to a significant reduction in the extinction of light by the nanoparticle. As the nanoparticle became more transparent, it underwent partial cloaking. The coupled system modified the radiative properties of the molecules. The efficient coupling depends on the distance between the molecule and the nanoparticle, and it is experimentally difficult to position the molecule at a specific location with respect to the nanoparticle. In the experiment, the researchers selected the molecule from a random distribution of dye molecules inside a crystal. This study opened quantum photonics applications through plasmonic coupling. Apart from coupling to microcavities or to plasmonic structures, a fluorescent molecule can be coupled to a dielectric waveguide (e.g., silicon nitride Si_3_N_4_ waveguides^18^ or gallium phosphide (GaP) waveguides^9^). This is promising for the development of quantum photonic chips and nanoscopic sensing of single charges.^9^

There has recently been a growing interest in cryogenic super-resolution microscopy^39,51^ and its combination with electron microscopy (specifically electron tomography).^52^ This new technique is powerful enough to obtain subcellular information on a specific biomolecular structural organization. The search for new host–guest systems has been continued. Schofield et al.^53^ reported a new guest–host system, DBT in *para*-terphenyl crystal, which allowed an easy and controlled sample preparation for nanocrystal growth.^54^ Moradi et al. investigated a new guest–host system, DBT in DBN discussed earlier, which demonstrated a strong linear Stark effect.^33^ Smit et al.^55^ recently reported the reverse intersystem crossing (rISC) of deuterated perylene in dibenzothiophene, which helped in recovering the fluorescence loss by the ISC process. Such a mechanism is promising for controlling the triplet state lifetime, specifically for the purpose of super-resolution imaging under cryogenic conditions.^38,56^ Apart from investigating the electronic states of a molecule, the study of a vibrational energy state and its higher-resolution spectroscopic investigation are important steps for quantum-optical applications. Zirkelbach et al.^57^ recently reported that high-resolution vibronic spectra can be experimentally obtained by combining single-molecule fluorescence excitation spectroscopy with stimulated emission depletion (STED) spectroscopy. They found several DBT molecules in a *para*-dichlorobenzene crystal that showed narrow lifetime-limited vibronic line widths of a few GHz, indicating longer lifetimes than those commonly admitted. Very recently, Orrit’s group^58^ reported the first observation of near-lifetime-limited ZPL of a single organic dye molecule on a substrate surface (hexagonal boron nitride (hBN) surface). They found ZPL line widths of single terrylene molecules of a few 100 MHz (Figure 3D–F). Future studies of this two-dimensional van der Waals matrix promise an alternative guest–host system as a single-photon source for integrated quantum nanophotonics. Vainer et al.^59^ reported that ultranarrow zero-phonon lines are sensitive to the electron–phonon coupling in disordered solids. They measured the temperature-dependent spectral broadening of single tetra-*tert*-butylterrylene (TBT) molecules in polyisobutylene and toluene at cryogenic temperatures due to the electron–phonon coupling arising from the quasi-localized low-frequency vibrational modes.^60^ Recently Naumov et al.^61^ demonstrated that cryogenic fluorescence excitation spectroscopy can be used to map a spatially heterogeneous refractive index distribution as a material characterization of solid crystals of naphthalene and hexadecane, as well as semicrystalline polyethylene. To image many molecules simultaneously with their fluorescence excitation spectra, they used a camera for wide-field imaging at each frequency of a narrow-band laser tuned over the spectral range of interest.^62^ Such hyperspectral imaging has great potential for mapping spatial heterogeneity in solid materials.

## Future Perspectives

Here we propose and discuss some perspectives on single-molecule cryogenic spectroscopy, which we anticipate to be promising and interesting.

### 1 Manipulations of the Triplet States of Single Molecules

**Figure 4:**
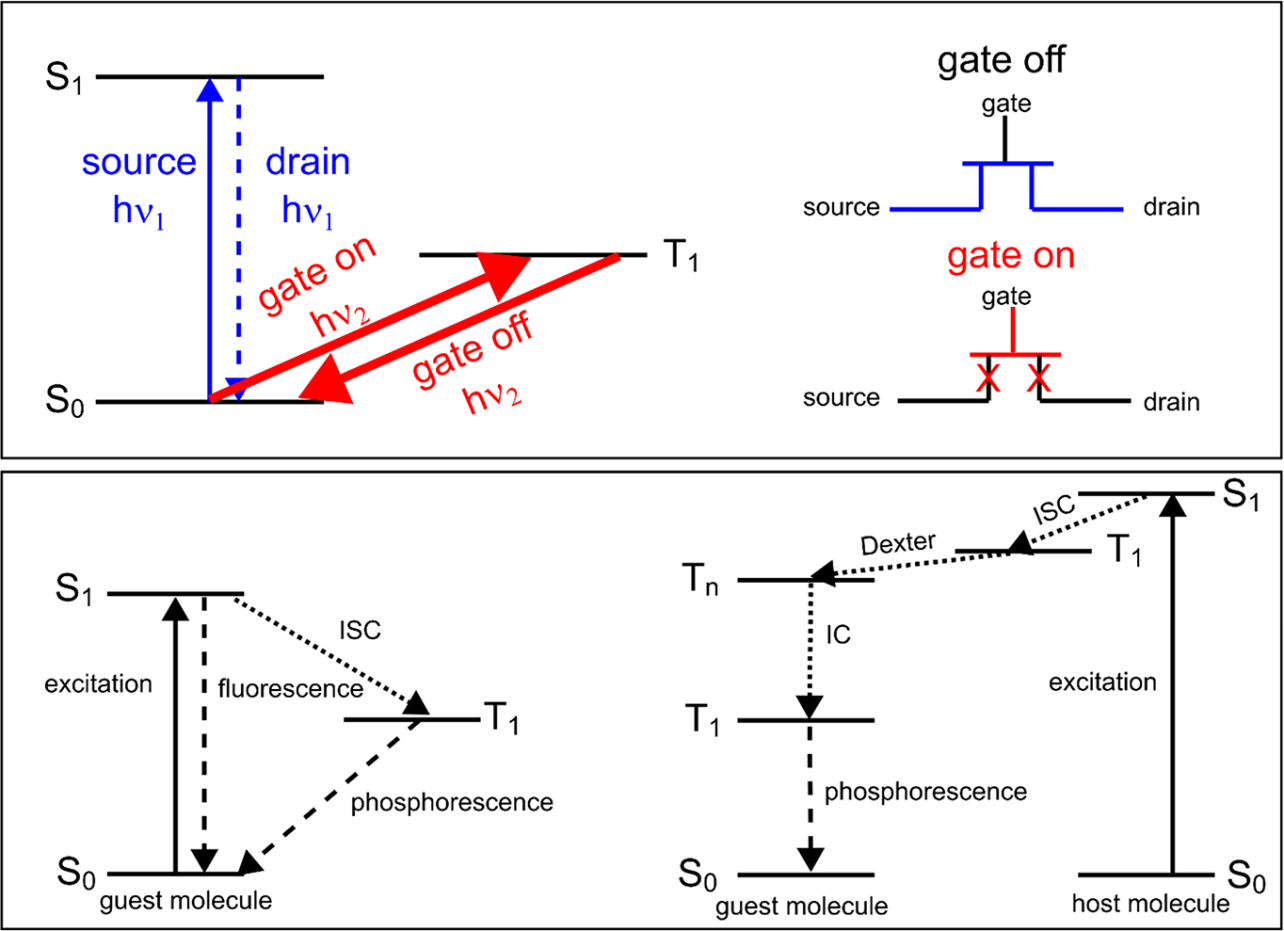
(Top panel) proposed mechanism for a single-molecule-based all-optical transistor. The weakly allowed transition from S_0_ to T_1_ with a strong resonant laser would block spontaneous transitions between S_0_ and S_1_. The transition back from T_1_ to S_0_ can be controlled by stimulated emission or depletion by the same strong resonant laser (or coherent pi pulses). The transition between S_0_ and T_1_ acts as a gate electrode, whereas the excitation and fluorescence transitions between S_0_ and S_1_ act, respectively, as the source and drain channels in the all-optical transistor (shown in the right). (Bottom panel) Proposed mechanism for finding the triplet states via phosphorescence by two mechanisms—(left) via intersystem crossing (ISC) and (right) via host-mediated Dexter energy transfer.

A single organic molecule can be used as a nanoscale all-optical transistor, which would allow miniaturization and the fast processing of quantum computing devices. The advantage of an optical transistor over an electronic transistor is that photons travel faster than electrons and photons, usually do not easily interact with each other, and are therefore easier to protect from decoherence than charges and spins. However, some type of photon–photon interaction is a prerequisite for an all-optical transistor. Single molecules, considered as three-level electronic systems (with ground singlet state S_0_, excited singlet state S_1_, and a metastable triplet manifold T_1_), can act as all-optical transistors if they can be transitioned from the S_0_ state to the long-lived T_1_ state via pumping with a strong resonant laser. Figure 4 schematically depicts a possible mechanism for an all-optical transistor. An attractive feature of singlet–triplet transitions, as compared to the previous demonstration of a single-molecule-based optical transistor,^46^ is the extremely high gain of the transistor, which can reach up to 10^6^ (considering the lifetime of a triplet state of milliseconds and the lifetime of a singlet excited state of nanoseconds).^14^ This considerable amplification has been first demonstrated with atoms^63^ but has so far remained out of reach of molecular systems because of lacking spectroscopic information about molecular triplet levels. The development of a fast and high-gain all-optical transistor consisting of only one organic molecule requires the determination of the energy of the triplet state’s energy.

Another attractive perspective of triplet manipulation is the study and manipulation of the nuclear spins of a single molecule. As we have discussed in the above section, ODMR experiments, combined with single-molecule fluorescence spectroscopy, showed for the first time that electron spins of a single molecule could be manipulated by using microwaves and that their magnetic resonance could be detected by measuring changes in the average fluorescence intensity. However, the manipulation of spins relies on the intersystem crossing, and therefore, one has to wait for the molecule to enter into the triplet state. Later on, the focus shifted to systems that possess spin in their ground state, such as NV centers,^64^ rare-earth ions, either embedded in solids^65^ or in molecules^66^ or some color centers,^67^ which could be resonantly excited on-demand. Through resonant excitation, these systems readily provided access to nuclear spins through hyperfine coupling. Nuclear spins are, in general, a promising resource for quantum memories as their coherence times can extend for up to seconds. For rare-earth ions, coherence times have been reached up to hours.^68^ Although the hyperfine interaction is necessary for the manipulation of nuclear spin, it also acts as a source of decoherence. Instead, a single organic aromatic molecule with a spinless ground state would be decoupled from hyperfine interaction after relaxation of the triplet necessary for the spin manipulation. With a resonant all-optical excitation of the triplet state of a single molecule, a single nuclear spin (e.g., that of a C-13 atom) could be manipulated optically. As mentioned above, resonant excitation of the triplet state requires knowledge of the triplet state energy. However, this spin-forbidden transition is extremely weak, with oscillator strengths as low as 10^–10^, and the energetic position depends sensitively on the chosen matrix due to solvent shifts. In this section, we propose some pathways for finding the energy of the triplet state in order to reduce the frequency range to be scanned for finding the narrow (width of about 1 MHz) resonance of the triplet.

Energies of triplet states have been detected in the past but never from molecules that have narrow and detectable resonances at low temperature and never at the single-molecule level. In ensemble experiments, the extinction of a laser beam by a transition from S_0_ to T_1_ has been measured, but this required the laser beam to pass through a very thick perylene crystal.^69^ In general, the transition to the triplet state via a direct excitation is very weak, and therefore, a more convenient way to locate the triplet state would be via detecting spontaneously emitted phosphorescence through the transition from T_1_ to S_0_. We will discuss two pathways for obtaining phosphorescence from guest molecules in a host matrix. The first pathway is via excitation of the guest molecules to their singlet excited state from which triplet states are populated by intersystem crossing (ISC). However, ISC is exceedingly weak for most molecules suited to fluorescence experiments. A second pathway is via excitation of the host matrix, followed by Dexter transfer to the triplet state of the guest. It is important to note here that both methods depend heavily on the phosphorescence yield of the guest molecules. For most molecules suited for single-molecule fluorescence spectroscopy, the quantum yield of phosphorescence is typically very low due to strong internal conversion. In addition, the quantum yield scales down considerably for molecules that have red-shifted emission which are often used in single-molecule experiments.^70,71^ Hence, among the standard single-molecule probes, the bluest emitter, perylene, is expected to have the best quantum yield of phosphorescence. This quantum yield can also be boosted by deuteration, which further reduces internal conversion.^72^ We therefore take perylene as an example for the following two cases.

For case (1), triplets are generated by stochastic intersystem crossing from the excited singlet state (Figure 4, bottom panel (left)). Therefore, the molecules have to be excited by the S_0_ → S_1_ transition, which for perylene has a wavelength between 440 and 450 nm. Once in the excited state, a small fraction of the excitations are converted to the triplet excited state,^55,73^ with a typical yield of about 10^–6^. The radiative quantum yield of the triplet would be expected to be around 10^–2^–10^–3^, thus reducing the yield of phosphorescence photons to 10^–8^–10^–9^. Such a low quantum yield would make it difficult to detect phosphorescence. The only matrix in which perylene phosphorescence was detected was anthracene.^74^ In this matrix, the close-by triplet of anthracene acted as an enhancer of the perylene triplet through intermolecular intersystem crossing.^75^ Unfortunately, the intermolecular ISC makes it very difficult to detect single molecules by fluorescence. The same authors^74^ attempted to detect phosphorescence of perylene in biphenyl but never managed to detect a signal. The enhanced rate due to intermolecular ISC made a big difference. Hence, case (1) is likely too difficult. Case (2) is when the phosphorescence of the guest is sensitized via Dexter energy transfer from the host (Figure 4, bottom panel (right)), and therefore, it relies on the host material for the formation of triplets, followed by energy transfer to the triplets of guest molecules. If a molecule (such as perylene) has a low ISC rate in a host matrix (such as *ortho*-dichlorobenzene^73^), the triplet state can be populated via the host-mediated transfer. A guest–host system with a low ISC rate is ideal for an all-optical transistor. Therefore, case (2) is more promising than case (1). The first step would be determining the triplet state energy level from an ensemble sample to obtain a strong enough phosphorescence signal. Once the triplet level is known from the ensemble measurement, the triplet state of a single molecule can be found by double resonance, scanning a narrow-band (∼MHz) red laser for the direct singlet–triplet transitions, measured by a change in fluorescence rate probed by a blue laser. It is important to mention here that any host–guest system should be free of impurities; otherwise, luminescence from impurities could hide the weak forbidden guest transitions.

### 2 Single-Electron Detection

Thanks to their narrow ZPL at cryogenic temperatures, single molecules can be sensitive probes of charges in their vicinity,^76,77^ which could be detected optically by a spectral shift of the ZPL induced by the electric field. In theory, single molecules are sensitive enough to detect single charges at a distance of up to 100 nm, such as those trapped in single-electron transistors or single-electron boxes.^78^ Contrary to single-electron transistors, which would require complicated fabrication techniques close to the structure to measure, crystals doped with single molecules are a versatile alternative for electrometry. One can stick, grow, or even drop cast (nano)crystals close to the region of interest and use each single molecule in them as an individual sensor, at temperatures up to 4 K. Although theory predicts that molecules can be ultimately sensitive to an external single electron, experimental evidence for this statement is still lacking and only limited works have demonstrated that molecules can detect a few charges in their vicinity, for example, in GaP waveguides,^9^ but the single-electron limit has not been reached yet. Single-electron charging within a molecule itself has been detected by significant spectral shifts that could be observed at room temperature through an internal Stark effect.^79^ The main problem for reaching the limit of single charges is that one needs a device that can control the amount of charge very well and operates at temperatures that are typically used in single-molecule spectroscopy: 1–4 K.

As we have discussed earlier, the insertion of DBT into DBN leads to a narrow distribution of Stark shifts around a maximum Stark shift of 1.5 GHz/kV·cm^–1^. In such a guest–host system, a single charge at a distance of 100 nm would induce an electrostatic field of at most 1.5 kV·cm^–1^, enough to shift a DBT molecules’ spectral line by many times its line width of about 40 MHz, if the net dipole moment is oriented parallel to the field. Using the spectral selectivity of single molecules and time-multiplexed measurements of their respective fluorescence signal, a single electron could be in principle triangulated and traced over time, as earlier suggested by Plakhotnik.^76,77^ The location of the molecules themselves can be found using cryogenic super-resolution techniques.

**Figure 5:**
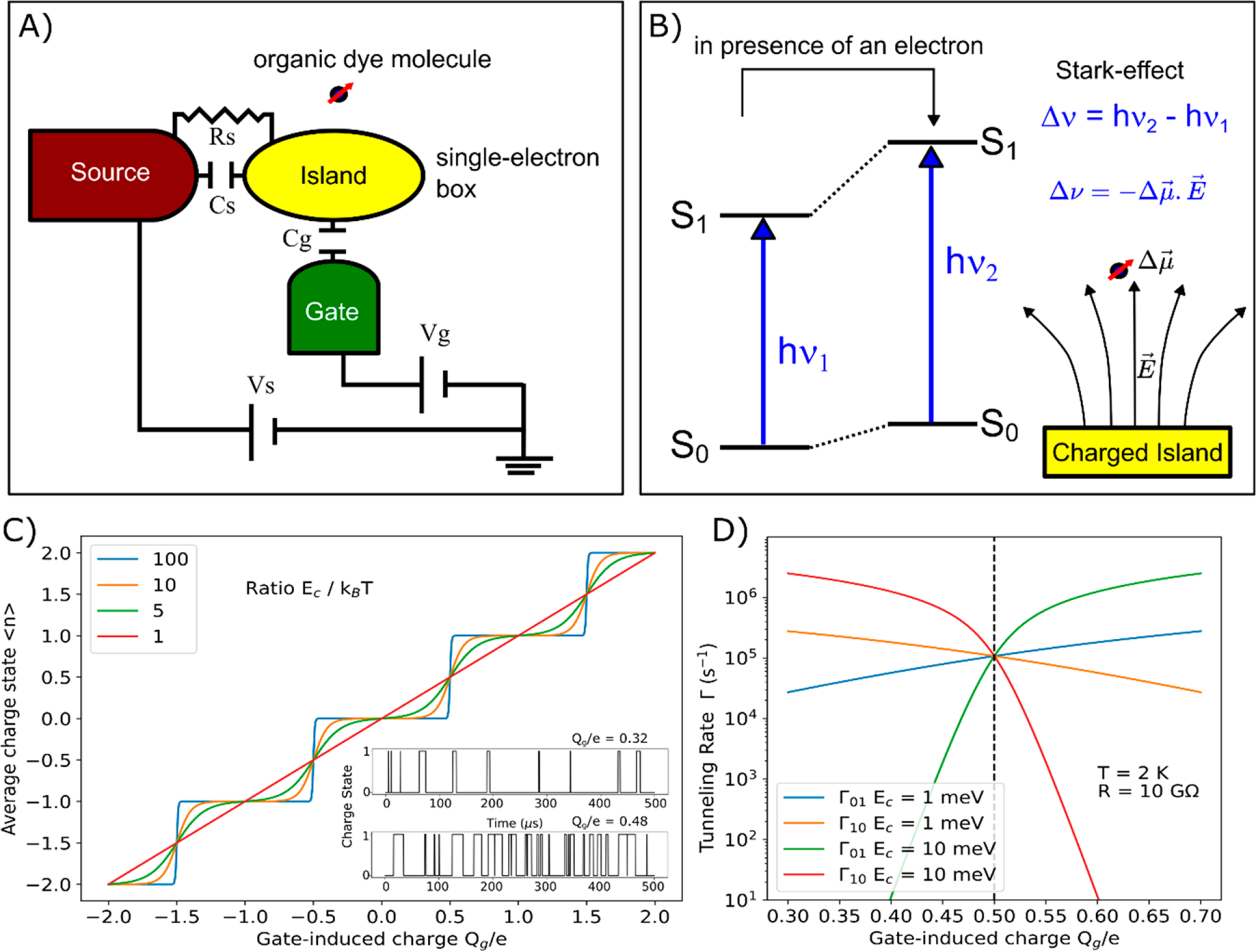
(A) Structure of the single-electron box. The gold island and source electrode are connected through a tunnel junction with a specified resistance *R*_s_ and capacitance *C*_s_. An additional gate electrode is capacitively coupled to the island with the capacitance *C*_g_. (B) Schematic that shows that the presence of an electron on the island shifts the electronic states of the close-by molecule through the Stark effect. (C) Expectation value of the island charge state as a function of the gate-induced charge (*Q*_g_ = *C*_g_*V*_g_ by an applied gate voltage *V*_g_, calculated by . The plotted curves correspond to different ratios of the charging energy to thermal energy. At a ratio of 100, the curve approximates a staircase, while, at lower ratios, the curve steps start to smear out. When the charging energy is close to the thermal energy, the charge state of the island is not well-defined. (D) Calculated tunneling rates, using eq 1 and 2, for a single-electron box with charging energies of 1 and 10 meV, at a temperature of 2 K and with a tunneling barrier of 10 GΩ. The parameters Γ_01_ and Γ_10_ respectively describe the tunneling rate for charging and discharging of the island. The tunneling rates are in balance at the charge degeneracy points or steps of the Coulomb staircase; e.g., *Q*_g_/*e* is 0.5, 1.5, and so on. Further away from the degeneracy points, the rates diverge and it will be harder to observe charge fluctuations. This is exemplified in the two graphs in the inset of (C), which show a simulated charge state of the island over time at *Q*_g_/*e* far away from the degeneracy point (*Q*_g_/*e* = 0.32) or close to it (*Q*_g_/*e* = 0.48). Far from the degeneracy point, the electron resides very shortly on the island and is thus difficult to detect experimentally. However, close to the degeneracy point, the charging and discharging rates are more similar. A higher charging energy does not slow down the rate at the degeneracy point but makes the charging step around the degeneracy point steeper, while the charging and discharging rates diverge more quickly further away from the degeneracy point. The tunneling rates scale down inversely with the tunnel barrier resistance, and above 1 GΩ, the rates will reach more practical values of below 1 MHz around the degeneracy point.

To demonstrate in a reliable manner that single molecules can detect single and multiple charges by the Stark effect, one needs a device that traps a controlled amount of charge for a long enough time. A textbook example of such a device is the single-electron box, which consists of a metallic island, with a typical size of 10–100 nm, and which is capacitively coupled to a source electrode and a gate electrode (Figure 5A and B), with a weak tunneling resistance between the source and the island. By adding a single charge from the source electrode to the metallic island, the electrostatic energy of the island increases by the so-called Coulomb or charging energy *E*_C_ = *e*^2^/2*C*_,_ where *e* is the electron charge and *C* is the box’s total capacitance with the environment. By reducing the island’s capacitance, the charging energy can exceed the thermal energy, *k*_B_*T* (*k*_B_ is the Boltzmann constant and *T* is the absolute temperature), and thus enter the regime of Coulomb blockade. In this regime, the charging energy will act as a barrier, reducing the rate of tunneling of single charges to and from the island, which is frequently characterized by the sequential tunneling model or “orthodox theory”, derived from Fermi’s golden rule:^80^
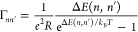
1Here, Δ*E* is the free energy, which is for the single-electron box given by
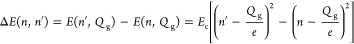
2Here, *Q*_g_ is the gate-induced charge, *Q*_g_ = *C*_g_*V*_g_, and *n* → *n*′ is the change of the charge state of the island (e.g., Γ_01_ and Γ_10_ are tunneling rates for, respectively, charging and discharging of the island). By tuning the potential of the island with the gate voltage, the barrier is reduced, and this modifies the tunneling rates forward and backward. Furthermore, the gate allows the control on the amount of charge on the island, typically measured as a Coulomb staircase, which as a function of gate voltage shows the expectation value of the number of charges on the island^81^ (Figure 5C).

Figure 5C shows that tuning the gate to some potential that changes the charge state does not necessarily lead to a well-defined charge state. Important for single-charge detection are the dynamics (such as that shown in the inset of Figure 5C, based on the tunneling rates shown in Figure 5D), or, in other words, the dwell times of the charge. If the dwell times are shorter than the excited state lifetime, the charge dynamics will likely contribute to dephasing, which may become apparent as a broadening of the zero-phonon line. For slower dynamics (ns to ms scale), the charge fluctuations could lead to spectral diffusion, line broadening, or line splitting, such as that observed for two-level systems, which could be studied using autocorrelations, as long as the dynamics are not too close to the time scales of photoblinking due to the triplet states. At even longer time scales (μs to s), the dynamics might be observed as quantum jumps in the resonance fluorescence of the molecule. Such slow regimes are difficult to achieve experimentally with typical single-electron boxes. However, these charge fluctuations have been measured as a random telegraph signal in μs–ms time scales in single quantum dots^82^ or quantum point contacts.^83^ These systems benefit from an additional quantization energy that is on top of the Coulomb barrier. Without additional quantization energy, similar charge dynamics have been achieved by adding extra islands to the single-electron box, which is called a single-electron trap. For the metallic single-electron box, the tunneling rates (eq 1) can be significantly slowed down by increasing the tunnel resistance. The barrier’s tunnel resistance could be well controlled by the growth of alumina layers by using atomic layer deposition. However, the best quality tunneling barriers could perhaps be achieved by using few-layer hexagonal boron nitride. Alternatively, the charging energy can be increased to an extent that the gate-induced charge on the island approximates a staircase, which may lead to clear steps in the molecule’s position caused by a sharp transition from *n* charges to *n* + 1 charges as the gate voltage is tuned. The charging energy can be increased by shrinking the island. Fabricating very small devices is a challenge, and therefore, in many single-electron devices, the operating temperature is reduced down to 10–20 mK, using dilution refrigerators.^82,83^ Typical experiments with single molecules use temperatures down to 2 K, but this factor of 100–200 in temperature makes a big difference. Another complication is that the single-electron box cannot be characterized electrically, although this would be possible by adding a drain electrode.

**Figure 6:**
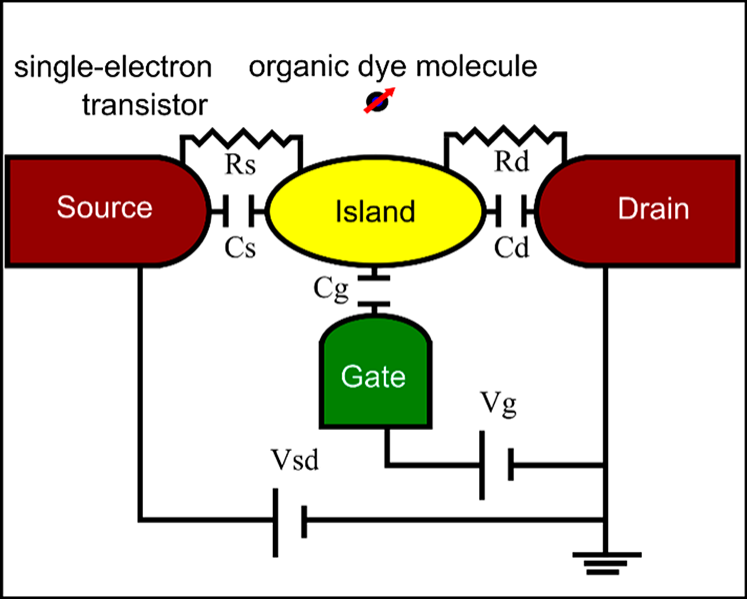
An extension of the scheme in Figure 5A. The inclusion of a drain electrode makes this a single-electron transistor. The drain electrode is capacitively and resistively coupled to the island and allows for the flow of current from source to drain.

An extension of the single-electron box would be combined with a drain electrode, i.e., the so-called single-electron transistor (see Figure 6). The addition of the drain electrode reduces the charging energy by another capacitive element. However, unlike the single-electron box it is possible to run a current through the device and do an electrical characterization, from which the charging energy can be extracted out of the *I*–*V* characterization.^84^ The tunnel barrier itself should not be too large, to make the current measurable, though this limits the possibility to observe real-time tunneling events. Typical tunnel barriers are up to a few MΩs. Given a temperature of 2 K, the charging energy should be at least 10–100 times larger or 2–20 meV to obtain well-defined charge states on the island. Such charging energies have been achieved using nanoparticle trapping,^85^ self-assembly of Au nanoparticles,^86^ or shadow evaporation.^87^

### 3 Plasmonic Enhancement at Cryogenic Temperatures

**Figure 7:**
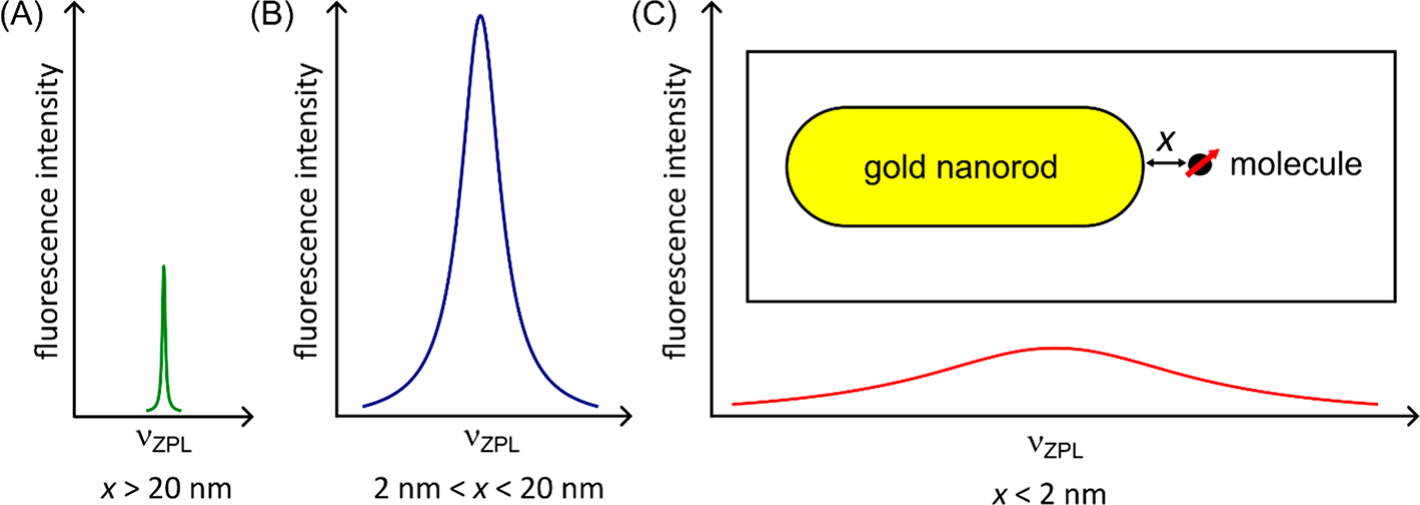
A scheme for studying plasmonic enhancement at cryogenic temperatures. (A) When a molecule is far from the near-field of a plasmonic nanorod, the fluorescence of the molecule has a lifetime-limited ultranarrow ZPL spectral line width. (B) When the molecule enters the plasmonic near-field, the fluorescence intensity at the ZPL is enhanced and the spectral line is broadened by the decrease in radiative lifetime. (C) When the molecule is very close to the nanorod, the molecule’s fluorescence is mostly quenched via non-radiative energy transfer, in addition to the reduction in radiative lifetime, and therefore, the ZPL spectral line width gets much larger. A schematic of a molecule positioned at distance *x* away from a gold nanorod is shown in the inset in (C). In such a configuration, the near-field is assumed to extend to about 20 nm. Many molecules in the near-field volume could be localized by a combination of position-dependent localization and spectral selection.

Single-molecule spectroscopy in general requires bright fluorescent molecules, so far limited to essentially aromatic molecules with high fluorescence yields. There are many more fluorescent dye molecules with low quantum yields. These molecules can also be detected individually if their fluorescence signal is enhanced by coupling to a plasmonic nanoparticle via near-field enhancement. Both the excitation and the emission of fluorescence are enhanced in the nanoparticle’s near-field by a combination of the lightning-rod effect close to tips and asperities of the particle and of resonant enhancement if the excitation and/or the emitted wavelength fall close to the plasmon resonance of the particle. The enhancement of the radiative emission rate is called the Purcell effect. The near-field fluorescence enhancement occurs within a few tens of nanometers from the plasmonic nanoparticle. However, at very close distances to the nanoparticle the fluorescence signal is partly quenched by the nanoparticle via non-radiative energy transfer. Room-temperature experiments mainly focus on molecules diffusing through the near-field volume to study the plasmonic enhancement.^88^ However, a quantitative comparison of observed plasmonic enhancements to theory requires good control of the position and orientation of the fluorescent molecule with respect to the plasmonic nanoparticle, which is naturally present in cryogenic experiments. Moreover, cryogenic experiments would give access to ultrashort fluorescence lifetimes down to picoseconds through spectral measurements in the frequency domain. A further advantage of cryogenic measurements is the possibility of addressing molecules both spatially and spectrally through their optical resonance. Thus, many more molecules could be studied in the near-field volume than under ambient conditions. Hereafter, we discuss the potential and challenges in the plasmonic enhancement of single-molecule fluorescence at cryogenic temperatures, as schematically illustrated in Figure 7.

To study near-field enhancement, nanoparticles have to be placed and located inside of a solid matrix. Scattering of nanoparticles may be difficult to distinguish from the background scattering by the solid matrix in which nanoparticles are embedded. Photoluminescence of plasmonic nanoparticles is a background-free, alternative detection method, which requires strong laser excitation because the luminescence quantum yield is very low (typically 10^–5^ for gold nanorods). Instead of a molecular crystal matrix, one could use hexagonal boron nitride (hBN) as a substrate and spin-coat the nanoparticles on top of the hBN surface. This design would minimize the scattering background. As the fluorescence enhancement depends on the orientation of the molecule, the orientation of the molecule with respect to the orientation of the nanorod would need to be determined through polarization-resolved measurements. Another important parameter in the interaction is the spectral overlap between the particle’s plasmon spectrum and the ZPL and vibronic transitions of the molecule. This spectral overlap could be varied by studying several molecules with ZPLs distributed in the inhomogeneous bandwidth. An early demonstration of molecule–plasmon coupling at low temperature has already been reported by Zirkelbach et al.^15^ On surfaces, a plasmonic effect has been induced by the tip of a scanning-tunneling microscope combined with excitation spectroscopy.^89^

### 4 New Guest–Host Systems

**Figure 8:**
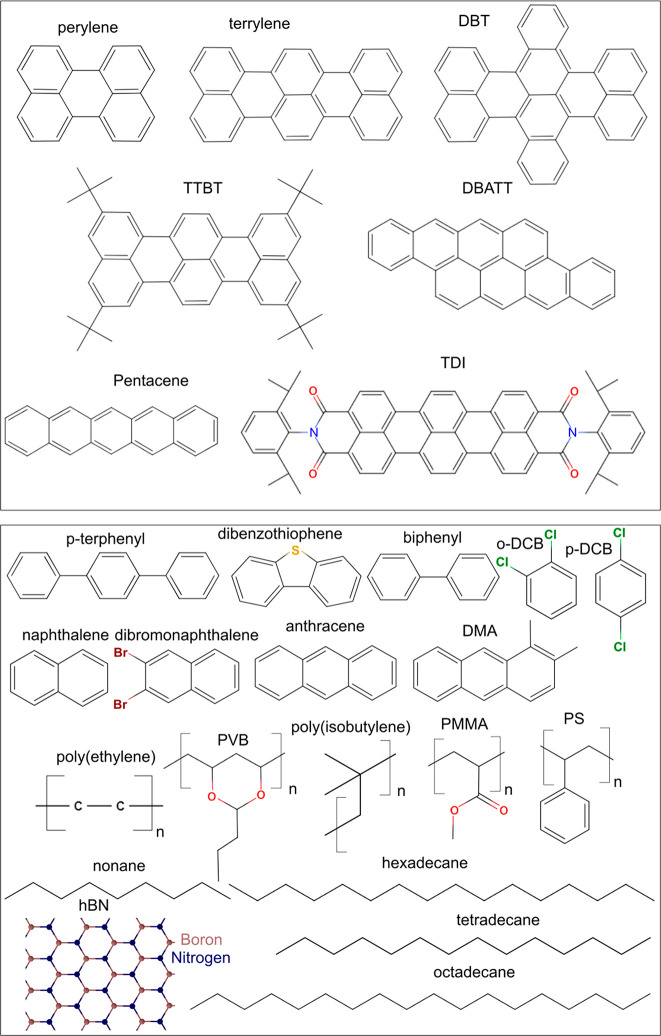
Chemical structures of some guest molecules (top panel) and host molecules (bottom panel). The structures are drawn using MolView online software.

After more than 30 years of research in cryogenic single-molecule fluorescence spectroscopy, only a small number of guest–host systems have been explored. We recall most of these systems in Table 1. One of the difficulties in exploring new guest molecules is finding the right host in which to embed them in. In most systems, dynamics take place even at low temperature and give rise to spectral instability (or spectral diffusion), which broadens the ZPL well beyond the lifetime-limit. Hereafter, we speculate on the origins of spectral diffusion and discuss how to match a host to a new guest molecule. A first parameter to consider is the size and shape of the host and guest. For example, the size mismatch between terrylene and anthracene^20^ (terrylene’s volume is about 2–3 times larger than anthracene’s) leads to the replacement of several host molecules by a guest upon insertion into the crystal. The mismatch may lead to many possible slightly different insertion geometries and, thereby, to spontaneous or light-induced spectral diffusion. However, details of the molecular shape are important. In *para*-terphenyl (similar in size to anthracene but with a different shape) terrylene molecules are stable, without spectral diffusion.^90^ Similarly, dibenzoterrylene, although similar in size to terrylene but with a slightly different shape, shows high spectral stability in anthracene.^26^ Therefore, size mismatch is not the only parameter to consider in the search for spectral stability. Conventional wisdom has it that rigid and well-crystallized aromatic host matrixes (e.g., naphthalene and anthracene) provide more photostability in comparison to more flexible host molecules (*p*-terphenyl and dimethylanthracene) or polymers. Table 1 indeed shows that in polymers molecules are spectrally unstable. Shpol’skii matrixes are layered crystals of *n*-alkanes (hexadecane, tetradecane, etc.), which in general provide good spectral stability as evidenced from Table 1. Host matrixes with flexible alkyl groups (such as methyl groups in 2,3-dimethylanthracene) allow rotational degrees of freedom which create a multidimensional energy landscape and thus promote spectral instability as reported in ref (91) for single terrylene molecules. Photoinduced or thermally induced spectral diffusion is probably unavoidable in complex systems.^92^ We speculate that the adsorption of planar aromatic molecules on 2D materials such as hexagonal boron nitride (hBN) or their embedding in van der Waals materials might greatly simplify the search for a suitable host. Terrylene molecules on a hBN surface showed spectral instability that was reduced upon prior annealing.^58^ Whether hBN can serve as a substrate or host for other guest molecules will be tested in future experiments. The encapsulation of molecules between hBN layers is an attractive method to protect guests from unwanted dynamics from surface contamination. Ideally, it could lead to narrower ZPLs at higher than liquid-helium temperatures due to the rigid structure and thus high phonon energies of this host. Although perylene, terrylene, and dibenzoterrylene span a spectral range between 450 and 780 nm, low-temperature single-molecule studies are limited to very few guest molecules compared to room temperature studies. Apart from the known guest molecules, unknown impurity emitters with narrow resonances have shown up in some experiments and are probably of aromatic origin. These emitters were found as impurities in solvents,^93^ in polymers/alkanes,^94^ and on hBN treated with toluene.^58^ Currently, the chemical structure of these emitters is still unknown, but perhaps the recently reported method of STM measurements combined with photoluminescence could aid in their identification.^89^ New promising classes of guest molecules with aromatic properties and a high fluorescence quantum yield, that could be part of future research, are synthesized graphene quantum dots.^95−97^ Host–guest systems and chemical structures are shown in Table 1 and Figure 8.

**Table 1:** ZPL Line Width, Spectral Stability, ZPL Spectral Position, and Fluorescence Lifetimes of Several Guest–Host Systems^a^

Guest	Host	Linewidth (MHz)	Spectrally Stable	λ_ZPL_ (nm)	τ_f_ (ns)	Refs.
Perylene	Polyethylene	∼100	No	∼450	∼6.3	(24)
Perylene-d12	Dibenzothiophene	∼58	Yes	∼454	∼4.6	(55)
Perylene	*o*-DCB	∼53	Yes	∼447		(73)
Perylene	Biphenyl	∼140	No	∼445		(74)
Perylene	*n*-Nonane	∼34	No	∼444		(98)
DPOT	*n*-Tetradecane	∼30	Yes	∼444	∼6	(99)
T1P4	Zeonex polymer	∼10^5^	No	∼530		(100)
PDI	PMMA	∼4000	No	∼528	∼3.9	(101)
Terrylene	Benzophenone	∼60	Yes	∼571	∼3.8	(102)
Terrylene	*n*-Alkanes	∼60	No	∼572	∼3.8	(20, 103, 104)
Terrylene	Anthracene	∼50	Yes	∼579	∼3.2	(75)
Terrylene	Polyethylene	∼80	No	∼569	∼3.8	(20)
Terrylene	PVB	∼600	No	∼562	∼4.4	(20, 103)
Terrylene	PMMA	∼1000	No	∼557	∼4.9	(20, 103)
Terrylene	PS	∼1700	No	∼566	∼4.8	(20, 103)
Terrylene	*p*-Terphenyl	∼50	Yes	∼578	∼3.8	(90)
Terrylene	Naphthalene	∼44	Yes	∼574	∼3.2	(22, 105)
Terrylene	*o*-DCB	∼60	Yes			(106)
Terrylene	2,3-DCN			∼580	∼3.5	(107)
Terrylene	*p*-DCB	∼45	Yes	∼572	∼4.2	(108)
Terrylene	Biphenyl	∼200	No	∼578		(109)
Terrylene	On hBN surface	∼450	Yes (annealed), No (non-annealed)	∼582	∼3.6	(58)
TBT	Poly(isobutylene)	∼100	No	∼567		(19)
TBT	Polyethylene	∼100	No	∼570	∼3.2	(19)
DPNP	*n*-Hexadecane	∼40	Yes	∼576		(110)
BDBP	*n*-Tetradecane	∼70	No	∼565	∼5.1	(21)
Pentacene	*p*-Terphenyl	∼20	Yes	∼593	∼25	(28)
Pentacene	*n*-Tetradecane	∼10	Yes	∼585	∼25	(111, 112)
PBI1	Hexadecane	∼55	Yes	∼602	∼3.5	(113, 114)
TDI	Polyethylene		No	∼659		(115)
TDI	Hexadecane	∼50	Yes	∼656	∼3.5	(115)
DBATT	Naphthalene	∼20	Yes	∼618	∼7.8	(49)
DBATT	Octadecane	∼20	Yes	∼589		(50)
DBATT	Naphthalene	∼20	Yes	∼618		(50)
DBATT	*n*-Tetradecane	∼17	Yes		∼9.5	(45)
DBATT	*n*-Hexadecane	∼15	Yes	∼589	∼9.4	(116)
DBATT	MMA	∼19	Yes	∼584	∼9.5	(117)
Porphycene	PVB			∼630		(118)
Graphene QDs	PS	∼0.8 nm (7 K)	No	∼650	∼5.0	(96, 97)
Molecule X	PMMA			∼605	∼3.8	(93)
DBT	*p*-DCB	∼23	Yes	∼744.7	∼7	(57)
DBT	*p*-Terphenyl	∼100	Yes	∼772	∼4.6	(53)
DBT	*p*-DCB	∼30	Yes	∼745		(9)
DBT	DBN	∼37	Yes	∼756	∼4.8	(33)
DBT	Anthracene	∼26	Yes	∼785	∼6.0	(26)
DBT	2,3-DMA	∼800	No	∼780		(91)

a Respective references are given. In the column “Spectrally Stable”, “Yes” indicates systems showing ZPLs with almost no spectral diffusion, whereas “No” indicates systems showing ZPLs with substantial spectral diffusions. PBI1, porphyrin–perylene bisimide–porphyrin; T1P4, polyphenylene dendrimer; PDI, perylene dimide; DPOT, diphenyloctatetraene; TBT, tetra-*tert*-butylterrylene; BDBP, benzodiphenanthrobisanthene; DPNP, 2.3.7.8-di-(peri-naphthylen)-pyrene; TDI, terrylenediimide; DBATT, dibenzanthanthrene; DBT, dibenzoterrylene; PVB, polyvinylbutyral; MMA, methyl methacrylate; PMMA, poly(methylmethacrylate); PS, polystyrene; *o*-DCB, *ortho*-dichlorobenzene; DMA, dimethylanthracene; hBN, hexagonal boron nitride; *p*-DCB, *para*-dichlorobenzene; DBN, dimethylnaphthalene; PS, polystyrene.

### 5 Other Future Perspectives

There have already been some reports on cryogenic super-resolution microscopy and its correlation with electron microscopy. In a way, similar to localization microscopy at room temperature based on stochastic photoblinking, Sandogdhar’s group has demonstrated super-resolution imaging at a cryogenic temperature with an Angström resolution.^38,39,51^ Lounis’ group developed three-dimensional nanoscopy based on excited-state saturation by illumination with a doughnut beam and obtained 30 nm axial resolution.^50^ One of the advantages in cryogenic super-resolution is the ability of a single molecule to emit a large number of photons, which allows the localization of a molecule with a very high precision. In addition, the fluorescent dyes used to label the biomolecules remain largely protected from photobleaching in the cold environment, so that even weak fluorescence signals can be correlated with cryo-EM.^52^ One of the limitations of cryogenic measurements is their lack of dynamical information, for example, about the conformational changes of a biomolecule. However, one can use temperature-cycle microscopy^119^ to obtain simultaneous high-resolution structural and conformational information.

Although the first single-molecule detection was based on an absorption signal, most single-molecule studies in later times were based on fluorescence. The lifetime-limited ultranarrow ZPL with a high absorption cross-section is able to extinguish focused light very efficiently. Single-molecule imaging has been reported based on the extinction signal; however, those studies focused on well-known single-molecule traditional molecules, such as DBT or DBATT. Single-molecule extinction imaging of many nonfluorescent molecules would broaden the applicability of the technique. Another imaging technique, which can be implemented for cryogenic single-molecule imaging, would be photothermal microscopy.^120^ Room-temperature photothermal microscopy has already demonstrated imaging of a single molecule’s absorption.^121^ One limitation for cryogenic photothermal microscopy is the low thermo-refractive coefficient of solid host matrixes. However, the absorption cross-section of a single molecule is much higher at cryogenic temperatures than that at room temperature. We anticipate that the low thermo-refractive coefficient could be compensated by the higher absorption cross-section. Another upcoming field is the combination of spectroscopy and STM imaging.^89^ With this method, both the spectral properties and electronic structure of a single molecule can be resolved. As mentioned before in section 4, this method could help identify unknown emitters.

Apart from the above perspectives, we expect that the applications of single molecules for single-photon sources and their implementation into integrated quantum chips will continue to be hot topics in the future.

## Conclusion

In this Perspective, we briefly review past and recent progress in cryogenic single-molecule spectroscopy, keeping our focus on some recent developments. At liquid-helium temperatures, single molecules can emit single indistinguishable photons whose coherent interactions are test benches for a large gamut of quantum-optical phenomena and for integrated nanophotonics. The zero-phonon line of a single molecule is Fourier-limited and presents a high intensity (or Debye–Waller factor) and thus can perform as an ultrasensitive probe for nanoscale dynamics such as local charge dynamics or molecular rearrangements. Two-photon interference, single-molecule transistors, cryogenic super-resolution, and many more experiments have become reality in the last few decades. Based on this impressive progress of cryogenic single-molecule spectroscopy, we have proposed some new perspectives including triplet state manipulation, single-charge detection, cryogenic plasmonic studies, and extensive searches for novel guest–host systems. Our hope is that many more researchers will come along into this ever-expanding field, where the best may be yet to come.
